# Kinetin induces microtubular breakdown, cell cycle arrest and programmed cell death in tobacco BY-2 cells

**DOI:** 10.1007/s00709-022-01814-6

**Published:** 2022-10-14

**Authors:** Andrzej Kaźmierczak, Ewa Siatkowska, Ruoxi Li, Sophie Bothe, Peter Nick

**Affiliations:** 1 Faculty of Biology and Environmental Protection, Institute of Experimental Biology, Department of Cytophysiology, University of Łódź, Pomorska 141/143, 90-236 Lodz, Poland; 2 Botanical Institute, Karlsruhe Institute of Technology, Fritz-Haber-Weg 4, 76131 Karlsruhe, Germany

**Keywords:** Callose, Cell cycle arrest, Kinetin, Microtubules, Programmed cell death, Tobacco BY-2

## Abstract

Plant cells can undergo regulated cell death in response to exogenous factors (often in a stress context), but also as regular element of development (often regulated by phytohormones). The cellular aspects of these death responses differ, which implies that the early signalling must be different. We use cytokinin-induced programmed cell death as paradigm to get insight into the role of the cytoskeleton for the regulation of developmentally induced cell death, using tobacco BY-2 cells as experimental model. We show that this PCD in response to kinetin correlates with an arrest of the cell cycle, a deregulation of DNA replication, a loss of plasma membrane integrity, a subsequent permeabilisation of the nuclear envelope, an increase of cytosolic calcium correlated with calcium depletion in the culture medium, an increase of callose deposition and the loss of microtubule and actin integrity. We discuss these findings in the context of a working model, where kinetin, mediated by calcium, causes the breakdown of the cytoskeleton, which, either by release of executing proteins or by mitotic catastrophe, will result in PCD.

**Supplementary Information:**

The online version contains supplementary material available at 10.1007/s00709-022-01814-6.

## Introduction

Although self-perpetuation represents a central (possibly the central) feature for living beings, death can occur not just as accidental by-product of damage or perturbation but can be initiated actively. This regulated form of cell death is, in fact, one of the most important processes controlling differentiation of plants (reviewed in Lam 2004; Locato and de Gara 2018), animals (Galluzzi et al. 2018) and of prokaryotic organisms (Tanouchi et al. 2013). The functions of regulated cell death (RCD), originally discovered in plants, are manifold — the removal of damaged cells, as to improve resource allocation to their intact neighbours, or the active self-elimination to provide material or signals for the development of other cells (McCabe et al. 1997). In wheat, the active self-elimination of epidermal cells as a very efficient way to ward off intruding hyphae from the rust fungus *Puccinia* (Allen 1923) was described, before so-called apoptosis became a buzz word in medicine.

The numerous functions of RCD have stimulated a plethora of different names of the process leading to terminological mess (Galluzzi et al. 2018; Locato and de Gara 2018). We will refer to those cases, where cells commit suicide as part of canonical development, as Programmed Cell Death (PCD) related with terminal differentiation of xylem vessels and sclerenchyma cells, or the breakdown of the suspensor during late embryogenesis as well as aerenchyma formation (Evans 2003). Instead, so called Exogenously Induced Cell Death (EICD) ensues as active (and often adaptive) response to biotic or abiotic stress factors. Typical examples would be the Hypersensitive Reaction (HR) of resistant hosts to biotrophic pathogens (Gong et al. 2019), or the salt-induced cell death of the root tip, which will stimulate the formation of lateral roots (Li et al. 2007).

Based on the morphologic and metabolic changes of the vacuole, at least two types of RCD can be discerned (for review see Lam 2004). In developmental PCD, a combination of autophagy and release of hydrolases from the eventually collapsed vacuole remove the cell content (van Doorn 2011), whilst during necrotic death occurring under severe abiotic stress, the plasma membrane loses integrity, which is preceded by shrinkage of nuclei and protoplast. The HR has also been referred to as mixed type because it seems can express features of both necrosis and vacuolar cell death (van Doorn et al. 2011). Since plant cells are encased in a rigid cell wall, their breakdown shows cytological features that differ from apoptotic death of animal cells. Also, the molecular details differ, because plants lack caspases, whose function is played by metacaspases and a couple of other proteases (for review see Piszczek and Gutman 2007). Moreover, the apoptotic bodies enveloped by plasma membrane, in which the animal cell is partitioned containing cytoplasm, nuclei, mitochondria and the endomembrane system, seem to be missing. However, comparable structures have been observed in plant cells as well and might help to degrade and recycle proteins, or even parts of entire organelles, during plant development or environmental stress. Again, two types of autophagy have been described (van Doorn and Woltering 2005): so-called microautophagy, where the tonoplast invaginates, such that cytoplasmic fractions are integrated into the vacuole and macroautophagy (reviewed in Bozhkov 2018), where a large phagophore, emerging from the endoplasmic reticulum (ER), first engulfs part of the cytoplasm and then releases its content into the vacuole for degradation. This autophagosome is surrounded by a double membrane, and the outer membrane fuses with the tonoplast. The regulation of the process depends on autophagy-related (ATG) proteins; some of which, such as the ATG1/ATG13, act as kinases on protein substrates, whilst others, such as the phosphatidylinositol-3-kinase (PI3K) complex, which converts phosphatidylinositol to phosphatidylinositol-3-phosphate (PI3P), seem to target to the membrane moiety.

To dissect the cellular details of regulated cell death and to link it with differential signal transduction requires a system, where cell death can be triggered by a signal. Whilst this has been done extensively for the HR, it is more difficult to address this for developmental PCD. Over the last decades, we have elaborated such a model. The cytokinin kinetin, widely known as a “hormone of life” for its stimulation of cell proliferation, can also act as “hormone of death” in particular situations. This hormone is able to initiate PCD within 2–3 days in parenchymatic cells of the apical root cortex in primary roots of *Vicia faba* ssp. *minor* (Kunikowska et al. 2013; Doniak et al. 2016), leading to the formation of an aerenchyma, a tissue rich in intercellular cavities allowing oxygen to reach the metabolically active cells in the meristem, even under conditions of waterlogging, drought or soil clogging, when diffusion is limiting (Evans 2003).

Extensive research on this model case for developmental PCD revealed the following features: (i) formation of small, later larger acidic lytic vacuoles; (ii) condensation of heterochromatin at concomitant decondensation of euchromatin; (iii) chromatin fragmentation mediated by exo/endonucleolytic enzymes; (iv) sealing of plasmodesmata in the cell walls of living cortex cells bordering the aerenchymatic cavities linked with clogging by callose; (v) thickening of cell walls in the bordering non-dying cells; and (vi) formation of micronuclei and/or apoptotic-like (pseudoapoptotic) bodies (Kunikowska et al. 2013; Doniak et al. 2014; Kaźmierczak et al. 2017). There are also (a) reduction in the number of mitochondria and their morphological malformations due to excessive formation of reactive oxygen species (ROS), (b) greater activity of the ROS scavenging enzymes and (c) increase in the total and cytosolic levels of Ca^2+^ ions in cortex cells (Doniak et al. 2016, 2017) and a decrease in steady-state levels of ATP (Kaźmierczak and Soboska 2018). The molecular features of kinetin-induced cell death include (1) unaltered protein amount, (2) fluctuating activities of H_1_- and core-histone kinases, (3) activation of serine- and cysteine-dependent proteases, (4) changes in *β*1 proteasome subunit activity, (5) leakage of potassium ions from roots, (6) loss of plasma and ER membrane potentials (manifest as reduced content of unsaturated fatty acids in the ER), (7) malformations of the nuclear envelope, (8) reduced content of total lipids and lipid peroxides, (9) reduced amount of phospholipids and alterations of their composition and (10) elevated amounts of cellulose, callose and other cell wall bound sugars (Kunikowska et al. 2013; Doniak et al. 2014, 2016, 2017; Kaźmierczak et al. 2017).

This plethora of responses leads to the question, what is cause and what is consequence, requiring either a temporal sequence or spatial structuring. The HR often initiates with the perception of pathogen effectors by specific nucleotide-binding-leucine-rich repeat (NB-LRR) receptors deriving from a co-evolutionary history of specific pathogens with their specific hosts (for review see Takken and Tameling 2009), activating specific members of metacaspases (Gong et al. 2019). For developmental PCD, perception and early signalling are not that clear. Perturbations in the integrity of the plasma membrane cause an activation of a NADPH oxidase, triggering remodelling of cortical actin filaments activating PCD (Eggenberger et al. 2017). For the regulated death of *Arabidopsis* cells in response to the cytokinin benzylaminopurine, the receptor CRE1/AHK4 is required (Vescovi et al. 2012). For tobacco BY-2 cells, phosphorylated cytokinins turned out to be active, which holds true both for isopentenyl adenosine (Mlejnek and Procházka 2002) and benzylaminopurine (Mlejnek et al. 2003). Whilst perception and signalling of developmental PCD and of HR seem to differ, certain downstream events linked with the execution of cell death might be shared. For instance, caspase-like activities play a role also for cytokinin-induced cell death as to be concluded from inhibitor studies (Mlejnek and Procházka 2002). Likewise, intracellular burst, either originating from perturbation of mitochondrial (for review see Balint-Kurti 2019) or from plastid (for review see Ambastha et al. 2015) electron transport, seems to be a common mechanism during the execution of both types of RCD. This would mean that at some point the initially separate signal chains must converge. A possible candidate for such a merging hub would be the cytoskeleton.

Microtubules and actin filaments are functionally and structurally interconnecting and show significant remodelling during early phases of RCD. However, cortical actin, subtending the plasma membrane, is rapidly depleted during HR (Chang et al. 2015), whilst the developmental PCD during vascular differentiation goes along with a specific bundling and elimination of cortical microtubules (Iakimova and Woltering 2017). Aerenchymatic cells as well as vascular bundles differentiate from parenchymatic precursors and undergo terminal differentiation in response to specific plant hormones, such as auxin and kinetin (Byczkowska et al. 2013), involving ROS and ethylene as well (Evans 2003; Kaźmierczak et al. 2021).

Cell suspensions of *Nicotiana tabacum* BY-2 (deriving from pith parenchyma that can generate aerenchyma and vasculature) can serve as convenient cellular model to study cell death in response to kinetin.

In the current work, we transferred kinetin-induced cell death in roots of *V. faba* ssp. *minor* to cell suspensions of *Nicotiana tabacum* BY-2 (deriving from pith parenchyma that can generate aerenchyma and vasculature) because these cells show vigorous cell proliferation (Huang et al. 2017) and are amenable to transformation, such that GFP-tagged strains allow for life-cell imaging. In the current work, we first show that kinetin-induced PCD can be recapitulated in tobacco BY-2 cells. We characterise this case of developmental PCD, using double staining with the membrane-permeable dye Acridin Orange and the membrane-impermeable dye Ethidium Bromide, visualisation and measurement of cytosolic calcium ions and of callose. We further use GFP-tagged marker lines for microtubules and actin filaments, and are, thus, able to follow the cytoskeletal response to kinetin. This allows us to observe that kinetin-induced cell death is heralded by an elimination of cortical microtubules, whilst actin filaments (whose remodelling is an early hallmark of HR) retained their integrity. These findings lead to the novel working model where the different elements of the cytoskeleton are involved in the different types of plant RCD.

## Materials and methods

### Cultivation of tobacco suspension cells

Different strains of tobacco BY-2 (*Nicotiana tabacum* L. cv Bright Yellow-2) suspension cells (Nagata et al. 1992) were used for this study. In addition to the non-transformed wild type, strain BY2-TuB6-GFP, expressing *β*-tubulin (AtTUB6) fused to GFP under the control of the constitutive Cauliflower Mosaic Virus 35S promotor (Hohenberger et al. 2011), and the strain FABD2-GFP, expressing the first actin-binding domain of fimbrin (AtFIM1) in fusion with GFP, also under a CaMV-35S promoter (Maisch et al. 2009) were employed. Cells were subcultured in Murashige-Skoog medium at weekly intervals, by inoculating 1.5 mL of stationary culture cells into 30 mL of fresh medium and cultivated at 26 °C in the dark under constant shaking an orbital shaker (IKA®KS 260 basic) as described previously (Maisch and Nick 2007).

The untreated controls were monitored on a daily base from the time of subcultivation (defined as day 0). Kinetin (50 µM) was added at day 3 after subcultivation. If not stated otherwise, the treated cells were followed from day to the end of the cultivation cycle at a daily base as well. The parameters measured are given below.

### Physiological and metabolic monitoring of the cell lines

To get insight into the physiological state of the cells, the lines were monitored over the culture cycle. To measure fresh mass (FM), aliquots of 1 mL of cell suspension were drained from medium using custom-made tubes with a nylon mesh with a pore diameter of 100 µm^2^ (Nick et al. 2000). The medium was collected in a reaction tube, and to ensure complete drainage, the cells were subjected to a stream of pressurised air from a manual tube. The collected cells were weighed, transferred to fresh reaction tubes and fixed with equal volumes of 5% glutaraldehyde in 200 mM sodium phosphate buffer (pH 7.4). These samples were stored at 4 °C for subsequent staining (see below). The volume of the drained medium was determined by weighing and expressed as mL^−1^. Then, this medium was used to analyse conductivity (as readout for cell leakage) in mS (milli-Siemens) using a conductivity meter (Elmetron, Poland) as described previously (Byczkowska et al. 2013). Furthermore, calcium and glucose concentrations (in mg L^−1^) were determined using colorimetric strips (RQflex 10 plus, Merck, Darmstadt) following the protocol of the manufacturer. Calcium ions react quantitatively with glyoxal bis(2-hydroxyanil) to form a red complex in the presence of hydrogen peroxide, whilst glucose is converted by glucose oxidase into gluconic acid lactone, which in the presence of peroxidase and the hydrogen peroxide reacts with an organic redox indicator to form a blue-green dye. The concentration of residual sucrose in the medium was determined by refractometry (HI 96,801, Hanna, Germany) as readout for the integrated energy consumption and expressed in % Brix.

### Estimation of cell number over particular phases of the cell cycle

Frequency distributions over the different phases of the cell cycle were constructed for non-transformed wild-type cells raised either under control conditions or in presence of 50 µM kinetin (Sigma-Aldrich, Deisenhofen, Germany) added at subcultivation. For this purpose, the nuclei were stained with 4′,6-diamidine-2′-phenylindole dihydrochloride (DAPI) according to Kaźmierczak (2010).

In brief, the cells were fixed (see the “Physiological and metabolic monitoring of the cell lines” section) and then the fixative was washed out three times with three volumes of 100 mM sodium phosphate buffer (pH 7.4), followed by three passages with three volumes of staining buffer (100 mM sodium phosphate buffer supplemented with 10% of 200 mM citric acid) using the custom-made staining chamber described above (see the “Physiological and metabolic monitoring of the cell lines” section). After the pre-equilibration, cells were stained with 2 μg mL^−1^ of DAPI in staining buffer for 15 min in the staining chamber inserted into small (5 mL) beakers before washing three times with three volumes of staining buffer void of DAPI. Aliquots of 50 µL of the stained cells were then viewed by fluorescence microscopy (Optiphot-2, Nikon, Japan) with the UV2A filter, and photographed using an ACT-1 digital camera (Precoptic, Poland). The fluorescence intensity of the nuclei, reflecting DNA content, was quantified from the digital images using the ImageJ software (https://imagej.nih.gov). Hereby, the channels of the RGB image were split using the “split channel” tool. The red channel was then thresholded such that the nuclei were highlighted. Using the tracing tool, each nucleus was then individually selected to measure its integrated density. Subsequently, frequency distributions over fluorescence intensity were constructed, which allowed to infer the stage in the cell cycle (Kaźmierczak 2003). Hereby, cells in *G*_1_ were estimated as proportion of cells with a fluorescence intensity below 100 a.u. added to half of those with intensities between 100 and 400 a.u., cells in *S* phase as proportion of cells with a fluorescence intensity between 400 a.u. and 600 a.u. divided by two, whilst the remaining cells were defined as being in *G*_2_. Data represent mean and standard errors from two independent experiments consisting of three technical replications with 600–800 individual nuclei per replication.

### Estimation of cell mortality by the Evans Blue Dye Exclusion assay

To assess the effect of kinetin on mortality, non-transformed BY-2 wild-type cells were followed over the entire cultivation cycle in response to 50 µM kinetin as compared to untreated cells. Mortality was scored by the Evans Blue Dye Exclusion Assay (Gaff and Okong’o-Ogola 1971) as described in Sarheed et al. (2020). Data represent three independent replications with a population of 600 individual cells per replication.

### Cytochemical characterisation by Acridin Orange/Ethidium Bromide staining

A double labelling with the membrane permeable dye Acridine Orange (100 µg mL^−1^) and the impermeable dye Ethidium Bromide (100 µg mL^−1^) in 200 mM sodium phosphate buffer at pH 7.4 were used to get more cellular details on the type of kinetin induced mortality. Aliquots of 500 µL of cells cultivated under control conditions and cells cultivated in presence of 50 µM kinetin were collected daily from day 3 after subcultivation until the end of the cultivation cycle into a custom-made staining chamber and processed as described in Sarheed et al. (2020). Aliquots of 50 µL of the double-stained cells were then inspected by fluorescence microscopy (Diaplan, Leitz) using excitation in the blue (filter set I3, excitation 450–490 nm, beam splitter 510 nm, emission filter > 515 nm). For each experimental set, around 350–400 cells were recorded by a digital camera (Leica DFC 500) controlled by a digital acquisition software (Leica Application Suite, v4). In this approach, the nuclei of cells with tight cell membranes will appear green, because they are exclusively labelled by Acridine Orange. With progressive permeabilisation of the membrane, the red signal from Ethidium Bromide will increase, such that the nuclei turn over orange into red. The resulting colours of the chromatin were quantified using the Scn Image quantitative image analysis software (https://scion-image.com) as described in Byczkowska et al. (2013). The resultant fluorescence intensity (RFI) allowed to classify the cells into different stages, exemplarily shown in Fig. 3B: living cells appeared green, cells in dying stage I exhibited a yellow nucleus, whereby the nucleolus remained unstained, whilst cells in dying stage II were characterised by yellow nuclei where the nucleolus was labelled as well, and dead cells could be recognised by their red colour. Data represent mean and standard errors from two independent experiments consisting of three technical replications with 350–400 individual cells per replication.

### Quantification of callose

Callose was visualised and quantified using Aniline Blue (Kaźmierczak 2008). Cells were fixed in 2.5% glutaraldehyde in 200 mM sodium phosphate buffer (pH 7.4), and then pre-equilibrated three times for 3 min with 4 mM K_2_HPO_4_ (pH 9), prior to staining for 15 min with 0.05% (w/v) Aniline Blue (Water Blue; Fluka) in 4 mM K_2_HPO_4_ (pH 9). Unbound dye was washed out thrice using the same buffer. Callose was then recorded by fluorescence microscopy (Optiphot-2, Nikon, Japan) upon excitation with short-wave blue light (390–420-nm excitation filter as a green to yellow fluorescence). Digital images were recorded (ACT-1 digital camera, Precoptic, Poland) and the callose-dependent Aniline Blue signal was quantified using the Scn Image quantitative image analysis software (https://scion-image.com) as described in Kaźmierczak (2008). Data represent mean and standard errors from two independent experiments consisting of three technical replications with 150–250 individual cells per replication.

### Quantification of cytosolic and nuclear calcium levels

Calcium was estimated in situ by chloro-tetracycline according to Doniak et al. (2016). After fixation in 2.5% glutaraldehyde in 200 mM sodium phosphate buffer (pH 7.4), the cells were washed three times for 5 min with 50 mM of staining buffer (Tris–HCl, pH 7.45) in the custom-made staining chamber. After draining, excess liquid was removed using with a filter paper, and the cells were stained for 5 min in 5-mL beakers with 100 μM chlorotetracycline in staining buffer. Unbound dye was washed out three times with staining buffer for 2 min and then cells were analysed by fluorescent microscopy (Optiphot-2, Nikon, Japan) recording digital images (ACT-1 digital camera, Precoptic, Poland) using filter set B2A. The green fluorescence was quantified using the Scn Image quantitative image analysis software (https://scion-image.com) as described in Kunikowska et al. (2013). Data represent mean and standard errors from two independent experiments consisting of three technical replications with 50 to 100 individual cells and nuclei per replication.

### Live-cell imaging of the cytoskeleton

We visualised microtubules by means of the *Arabidopsis thaliana β*-tubulin (*AtTuB6*) marker, carrying a N-terminal fusion with GFP (Hohenberger et al. 2011), and actin filaments by means of the second actin-binding domain of *A. thaliana* fimbrin (*AtFIM1*) in fusion with GFP (Maisch et al. 2009). We captured images by spinning-disc confocal microscopy using a CCD camera on an AxioObserver Z1 (Zeiss, Jena, Germany) through a 63 × LCI-Neofluar Imm Corr DIC objective (NA 1.3), exciting with the 488-nm emission line of an Ar-Kr laser and collecting the signals through a spinning-disc device (YOKOGAWA CSU-X1 5000). To operate, we used the ZEN 2012 (Blue edition) software platform and generated orthogonal projections from the recorded stacks, exporting the raw images as TIFF format. For each experimental set, representative images of at least three independent experimental series recording a population of 30 individual cells were selected.

### Correlation analysis

In order to test correlations between different traits, we used a two-step procedure. In the first step, we tested the statistical significance for each trait individually by means of the Mann–Whitney *U* test and/or the Student’s *t*-test, using a threshold at *P* ≤ 0.05. In the second step, we plotted pairwise the respective regression curves and determined the ***r***_***xy***_ values. When |***r***_***xy***_| was between 0.0 and 0.3, we considered this as lack of correlation; values between 0.3 and 0.8 indicated a moderate correlation, and values above 0.8 a strong correlation. We visualised the result of this correlation analysis using yEd Graph Editor 3.20 (https://www.yworks.com/products/yed, open source).

## Results

### Kinetin delay growth, calcium uptake and glucose consumption

**Fig. 1:**
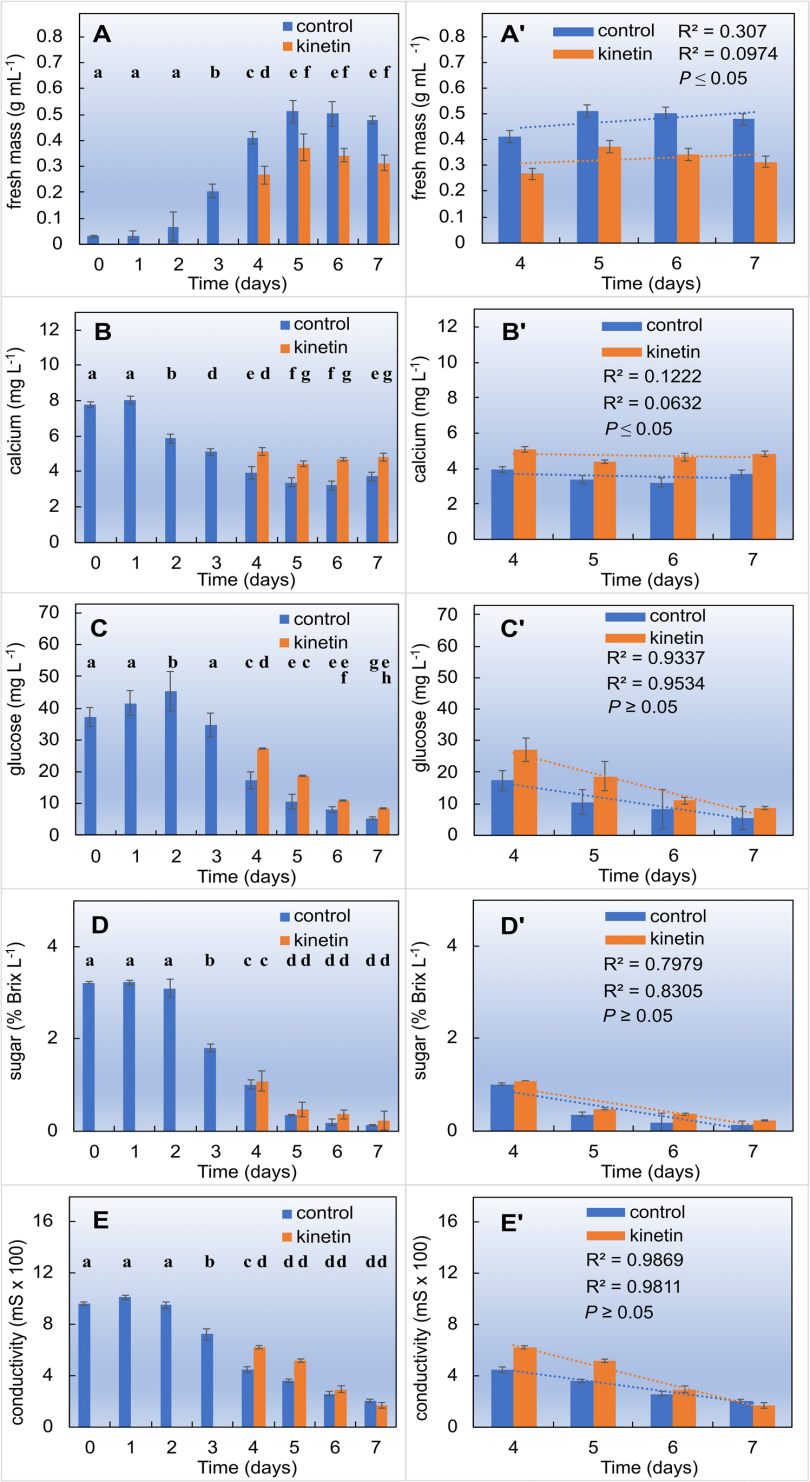
Physiological responses of tobacco BY-2 cells to kinetin (50 µM), added at day 3 after subcultivation. Time courses of **A** fresh weight of cells, **B** calcium, **C** glucose, **D** sucrose concentration in the medium and **E** conductivity of the medium, and their respective curve regression, *R*^2^ values and ***P ***(**A′–E′**) indicating statistical significance between Ctrl and Kin series are shown. Data represent mean values and SE from three independent biological replications

To monitor the physiological state of the cells in response to kinetin, we followed growth and metabolic parameters during the culture cycle. FM of control cells, after a lag of about 1 day, increased almost linearly until day 5, when it reached a plateau maintained until the end of the culture cycle (Fig. 1A). The addition of 50 µM kinetin at day 3 slowed down this increase, whilst the transition to the plateau occurred at day 5 as in the control. As a result, the plateau was reduced by about 30% compared to the control. This data is confirmed by determination values (*R*^2^) of the regression curves between the results from 4 to 7th days of culture (Fig. 1A′) that effect of kinetin is statistically different from control (*P* ≤ 0.05).

The concentration of calcium ions in the medium (Fig. 1B) displayed basically a mirror image of FM. After a lag of 1 day, calcium concentration decreased from the initial about 80 to around 40 mg L^−1^ from the 4th day. In the kinetin-treated samples, calcium concentration was constant throughout the interval between days 4 and 7 (*P* < 0.05) and around 20% higher than in the controls. Interestingly, the decrease of calcium concentration came to a halt at day 4, whilst FM (Fig. 1A) increased further until day 5. Thus, on the base of cellular FM, the uptake of calcium seems to decrease after day 4, when the cells undergo rapid expansion (Huang et al. 2017). This data is confirmed by determination values (*R*^2^) of the regression curves between the results from 4 to 7th days of culture (Fig. 1B′) that effect of kinetin is statistically different from control (*P* ≤ 0.05).

Glucose concentration in the medium (Fig. 1C) increased slightly (by about 10%) during the first 2 days of the culture cycle, reaching about 45 mg L^−1^; then glucose decreased steeply by about a factor of 8 reached at the end of the culture cycle. In the kinetin-treated cells, glucose concentration was about 60% higher compared to the control but decreased as well (from about 27 mg L^−1^ at day 4 to around a third at day 7). This decrease was more pronounced, such that at day 7, the glucose levels were almost as low as in the control. It should be noted that the medium was void of glucose. Thus, the glucose measured in the medium most derive from sucrose breakdown. In fact, total sugars, after an initial lag during the first 2 days of the culture cycle, decreased even more drastically, by a factor of 20 until the end of the culture cycle (Fig. 1D). Here, there was no significant difference between the kinetin-treated sample and the controls (*P* ≥ 0.05) what was confirmed by determination values (*R*^2^) of the regression curves (Fig. 1C, D′).

The time course of conductivity of the culture medium (Fig. 1E) was similar to that of glucose. After an initial lag phase of 2 days, it dropped from 1000 to around 200 µS at day 7. Again, in the kinetin-treated cells, conductivity was around 30% higher at day 4, but dropped than to the same level as in the control at day 7. Thus the there was no significant difference (*P* ≤ 0.05) between the kinetin-treated sample and the controls by comparison of determination values (*R*^2^) of the regression curves (Fig. 1C, D′).

This physiological mapping indicates that kinetin treatment delays culture growth, linked with a reduced uptake of calcium into the cells, a higher concentration of glucose in the medium and a higher conductivity. Instead, kinetin did not modulate the consumption of total sugars (the medium contained sucrose as carbon source).

### Kinetin induces arrest of the cell cycle and cell death in cycling cells of tobacco BY-2

**Fig. 2:**
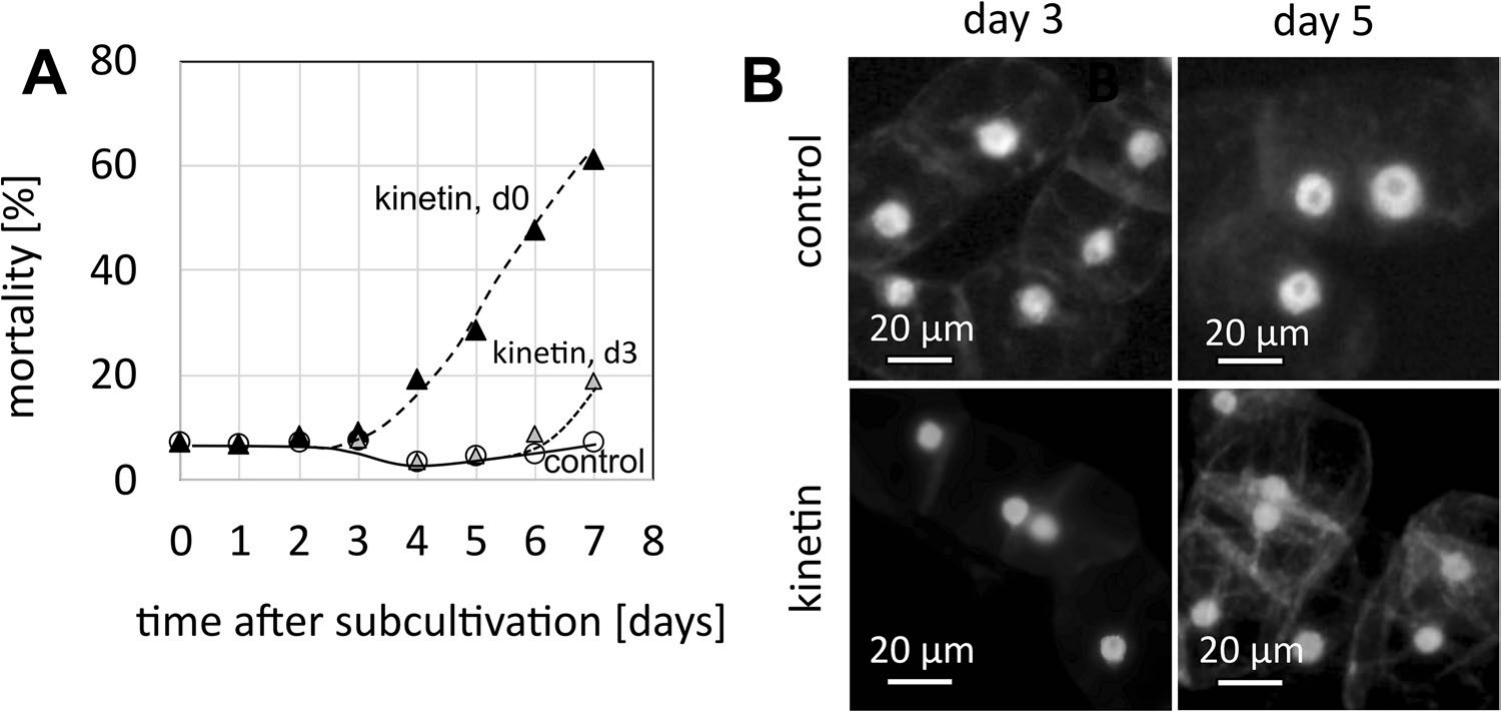
Cellular response of tobacco BY-2 cells to kinetin (50 µM), added at day 3 after subcultivation. **A** Time course of mortality for kinetin treatment from day 0 (Kin, d0), or from day 3 (Kin, d3) after subcultivation as compared to the untreated control (con). Data represent mean value from three independent experimental series scoring 600 individual cells per replication. **B** Representative cells stained with 4′,6-diamidine-2′-phenylindole dihydrochloride (DAPI) and viewed either at the peak of mitotic activity (day 3 after subcultivation) or at the time of maximal cell expansion (day 5 after subcultivation) either in control cells or cells treated with kinetin from day 0

To get insight into the effect of kinetin on proliferating cells, 50 µM of kinetin was either added at time of subcultivation or at the transition from cell proliferation to cell expansion. The nuclei were stained with DAPI, and mortality was scored by the Evans Blue Dye Exclusion Assay. Both readouts were followed over the entire cultivation cycle. In response to 50 µM kinetin administered at subcultivation, mortality remained low in the beginning equalling that seen in the untreated control (Fig. 2A). However, following day 3, mortality increased at a steady pace, reaching 60% at the end of the cultivation cycle at day 7. In contrast, mortality in the untreated control remained low. When kinetin (again 50 µM) was added during the proliferation phase at day 3, the increase of mortality was correspondingly shifted to day 7. Since it is not possible to prolong the cultivation cycle beyond 7 days, because otherwise tobacco BY-2 undergo cell death, only the initial phase of this mortality response could be captured. The value seen at day 7 was exactly that seen at day 4, when kinetin was added at subcultivation. Thus, the time course was just shifted by 3 days, meaning that kinetin induces a cell death response that requires 3 days to become manifest. Therefore, this cell death cannot be of an acute nature, but obviously requires cellular activities that need several days to unfold. To get insight into cellular aspects of the kinetin response, nuclei were stained with 4′,6-diamidine-2′-phenylindole dihydrochloride (DAPI) at different time intervals after subcultivation, either in untreated controls or in cells that had been treated with 50 µM kinetin from the time of subcultivation. When the cells were viewed at day 3 (around the peak of mitotic activity), the controls displayed large nuclei that were mostly located in the cell centre (Fig. 2B), characteristic for cells in *G*_2_. In contrast, cells that had been raised in presence of kinetin showed distinctly smaller nuclei that were often located close to the cell wall, which is characteristic for cells in *G*_1_. Two days later, at the peak of cell expansion, the nuclei in the kinetin-treated cells had increased in size and some were also seen in the cell centre, but still, they were significantly smaller than those of the control cells at this time point. Only at the end of the cultivation cycle, nuclear size in the kinetin-treated samples approached those of the control (Suppl. Fig. S1).

**Fig. 3:**
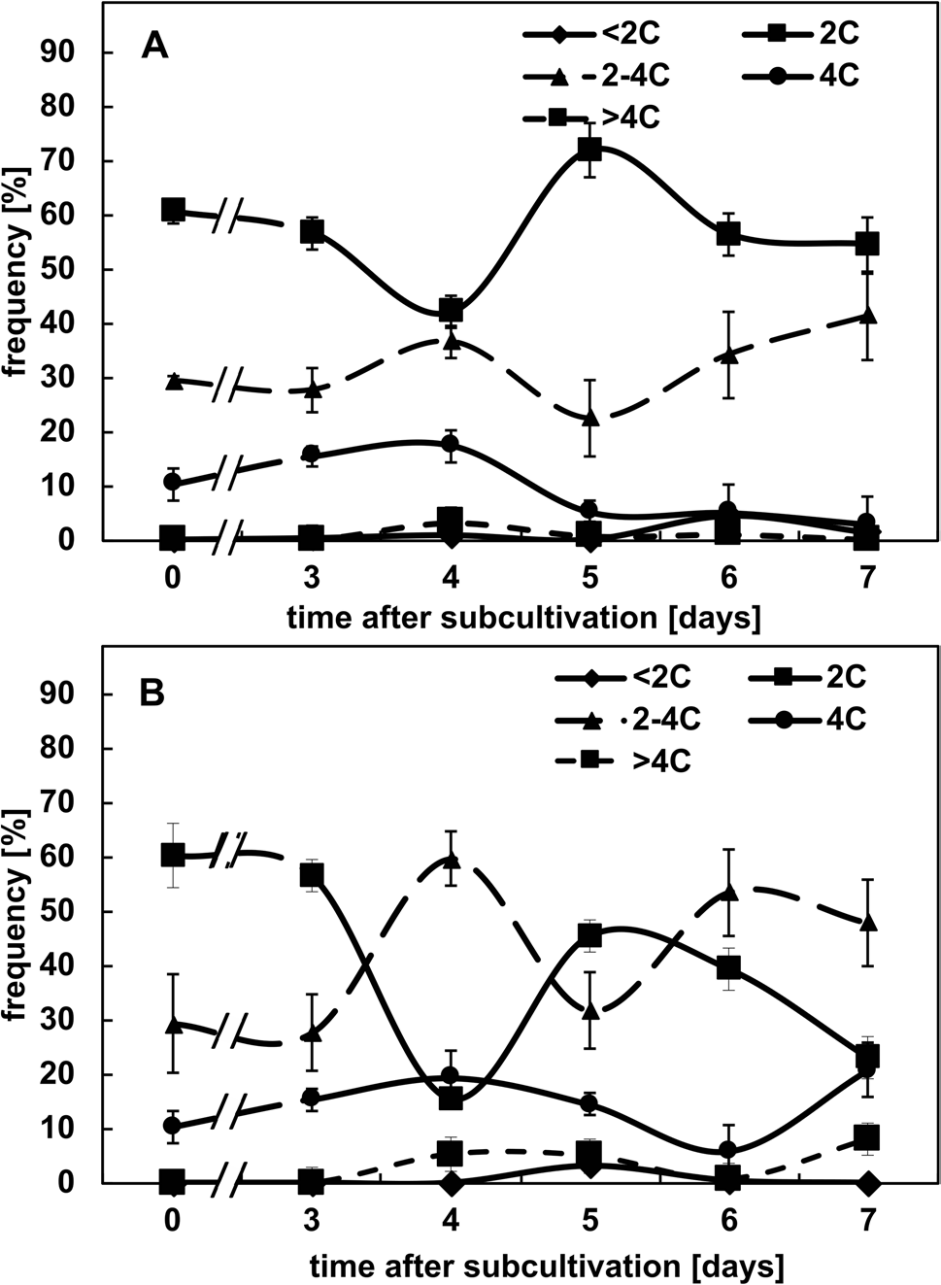
Frequency distributions over DNA content per nucleus inferred from DAPI staining through the cultivation cycle in control cells (**A**) and in cells that had been treated with 50 µM kinetin from day 0 (**B**). The DNA contents (C) were classified into hypoploid cells (< 2C), cells in *G*_1_ phase (2C), cells in *S* phase (2–4C), cells in *G*_2_ phase (4C) and endopolyploid cells (> 4C). Data represent mean and standard errors from two independent experiments consisting of three technical replications with a 600–800 individual nuclei per replication

To corroborate the impression that kinetin arrests the cell cycle, we estimated the DAPI signals from the individual nuclei by quantitative image analysis (which allows to infer DNA content) and constructed frequency distributions over this inferred DNA content through the cultivation cycle (Fig. 3). For the untreated control at subcultivation (i.e. at day 0), most cells (60%) displayed a DNA content of 2C, indicating that they were in the *G*_1_ phase of cell cycle (Fig. 3A). In contrast, cells in *S* phase (2–4C) were less frequent (around 30%), and those with completed *S* phase (4C) were even rarer (around 10%). At the time of maximal proliferation (between days 3 and 4), the incidence of cells in *G*_1_ phase had dropped to around 40%, whilst cells in or after *S* phase showed a concomitant increase (in the sum around 55%). During the subsequent phase of cell expansion, cells in *G*_1_ accumulated to around 70%, whilst the frequency cells in *S* or *G*_2_ dropped to a minimum (around 25% when cells with 2–4C and 4C are pooled). During the final phase of the cultivation cycle, more cells entered *S* phase, such that the values for cells in *G*_1_ dropped again to the initial level of around 60%. Thus, the dynamic fluctuation of DNA content per nucleus reflects a pattern, where cells enter a phase of rapid cycling that culminates between days 3 and 4. During the subsequent expansion phase, most cells remain arrested in *G*_1_. During the end of the cultivation cycle, they move on into the *S* phase, in anticipation of the next round of proliferations that are triggered by addition of fresh medium (which will also replenish cellular auxin levels).

The pattern observed for kinetin-treated cells was clearly different (Fig. 3B). During the first half of the cultivation cycle, the frequency of cells in *G*_1_ (2C) dropped much sharper than in the control, and whilst increasing again after day 4, it did not return to the initial value of 60%, seen in the controls, but dropped again to around 20% at the end of the culture cycle. Instead, the initial increase of cells during *S* phase (2–4C) or with completed *S* phase (4C) increased more dramatically (in the sum reaching up to 80% at day 4, which is significantly higher than the around 55% found in the control). Although the incidence of cells in *S* phase dropped subsequently, overall, the cells remained at significantly higher levels in *S* phase or *G*_2_ as compared to the control. This was also reflected in a shift of the intensity histogram towards higher intensities (Suppl. Fig. S2). These patterns support a scenario, where kinetin arrests the cell cycle in *S* phase and, thus, represses cell division.

### Cell death induced by kinetin shows cytological features of regulated cell death

**Fig. 4:**
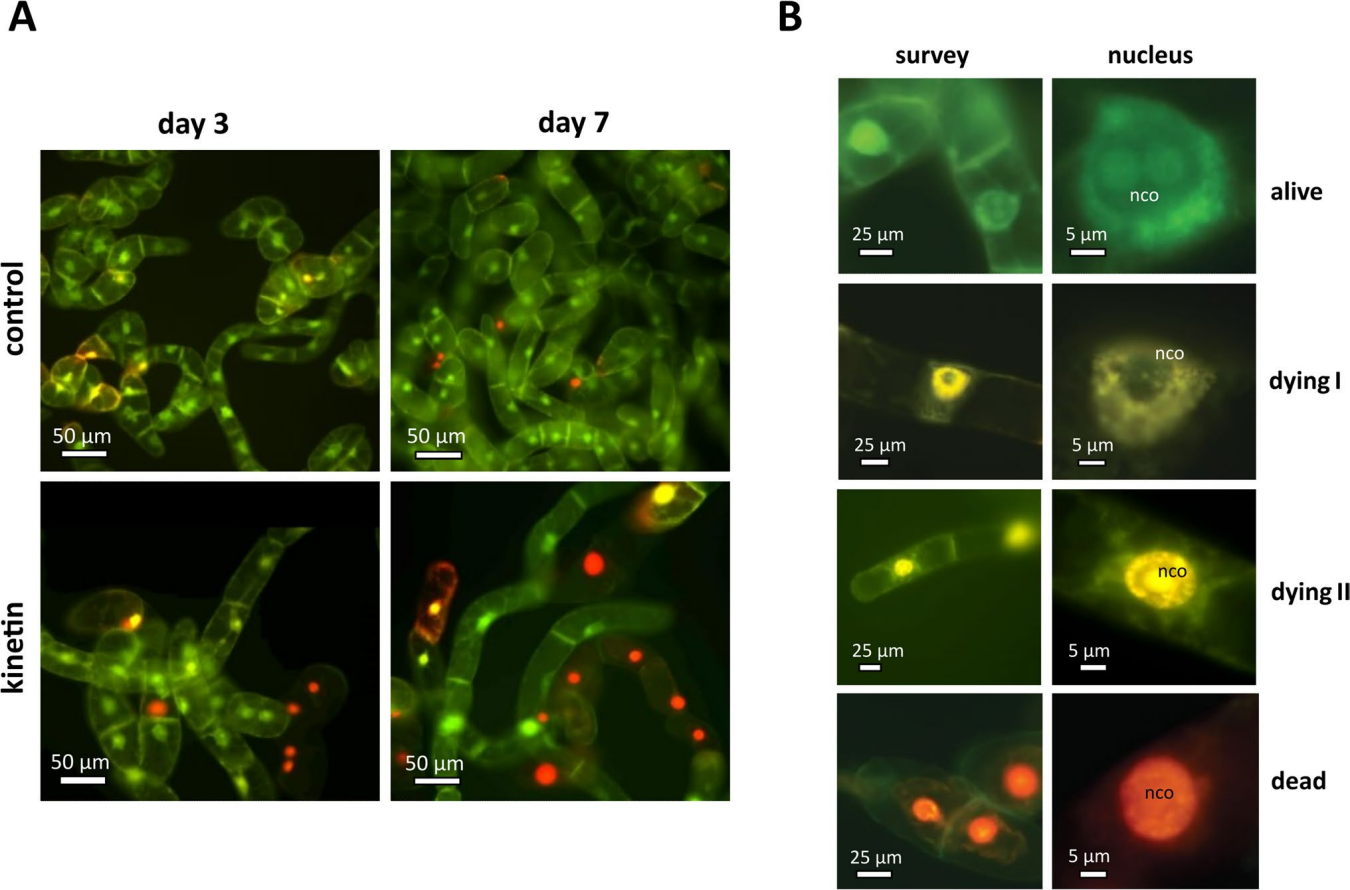
Appearance of BY-2 after double staining with Acridine Orange (membrane permeable green signal) and Ethidium Bromide (membrane impermeable, red signal) in untreated controls, or addition of 50 µM kinetin at the time of subcultivation. **A** Overview of the cells at day 3 or 7, respectively, merging the signals from the green and red channels. **B** Cellular details of the staging system used for classification in Fig. 5. *nco* nucleolus

The cell death in response to kinetin might be of an accidental nature, for instance, when the cellular processes activated by this hormone would exhaust vital functions of the cells. However, the resulting cell death might also be regulated. To differentiate between these two cases, we used a histochemical approach, staining the nuclei with two fluorescent dyes that differ with respect to membrane permeability. Acridine Orange is membrane permeable, whilst Ethidium Bromide is not. As long as the cell membranes are tight, the cells will appear green, whilst cells with a loss of membrane integrity will display nuclei that progressively change from orange into red. Whilst most control cells appeared green even at day 7 of the cultivation cycle (Fig. 4A, top row), kinetin treatment clearly increased the incidence of cells with red nuclei already from day 3 after subcultivation, indicative of a loss of membrane integrity (Fig. 4A, bottom row). A closer look reveals that the transition from the cells with intact membrane integrity (green cells, classified as “alive”) to those with a red nucleus (classified as dead) occurs through transitions, where both labels are present (Fig. 4B). These cells appear yellow. Most of these yellow cells, classified as “dying I”, display yellow nuclei with unstained nucleoli (Fig. 4B; *nco*); a smaller fraction of these yellow cells, classified as “dying II”, show yellow nuclei, where the nucleoli were lighting up massively as well. Obviously, in these cells, a permeability barrier still delineating the nucleolus during the stage dying I has broken down.

**Fig. 5:**
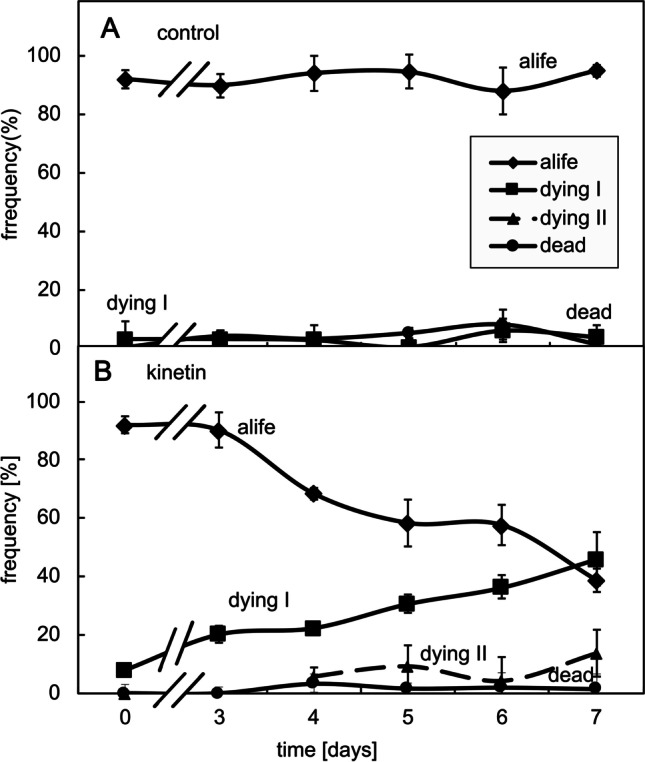
Frequency distributions over different stages of cell death as classified by double staining with Acridine Orange and Ethidium Bromide through the cultivation cycle in control cells (**A**) and in cells that had been treated with 50 µM kinetin from day 0 (**B**). The classification followed the system shown in Fig. 3B. Data represent mean and standard errors from two independent experiments consisting of three technical replications with 350–400 individual cells per replication

In the next step, we used this staging system to quantify the cellular response to kinetin over time (Fig. 5). In the control, almost the entire population was alive, exclusively exhibiting the green signal from Acridin Orange (Fig. 5A). In contrast, for the cells cultivated in the presence of 50 µM kinetin, the frequency of this class decreased progressively to less than 40% at day 7 (Fig. 5B). Concomitantly, the frequency of cells in stage dying I increased steadily, and even exceeded that of living cells at the end of the culture cycle. From days 3 and 4, also a minor fraction of cells in stage dying II (up to 15% at day 7) appeared. Interestingly, the frequency of dead cells remained low (below 5%) even at the end of the culture cycle. This indicates that dead cells are quickly degraded, such that their steady-state level remains low.

### Intracellular calcium levels are elevated in response to kinetin

**Fig. 6:**
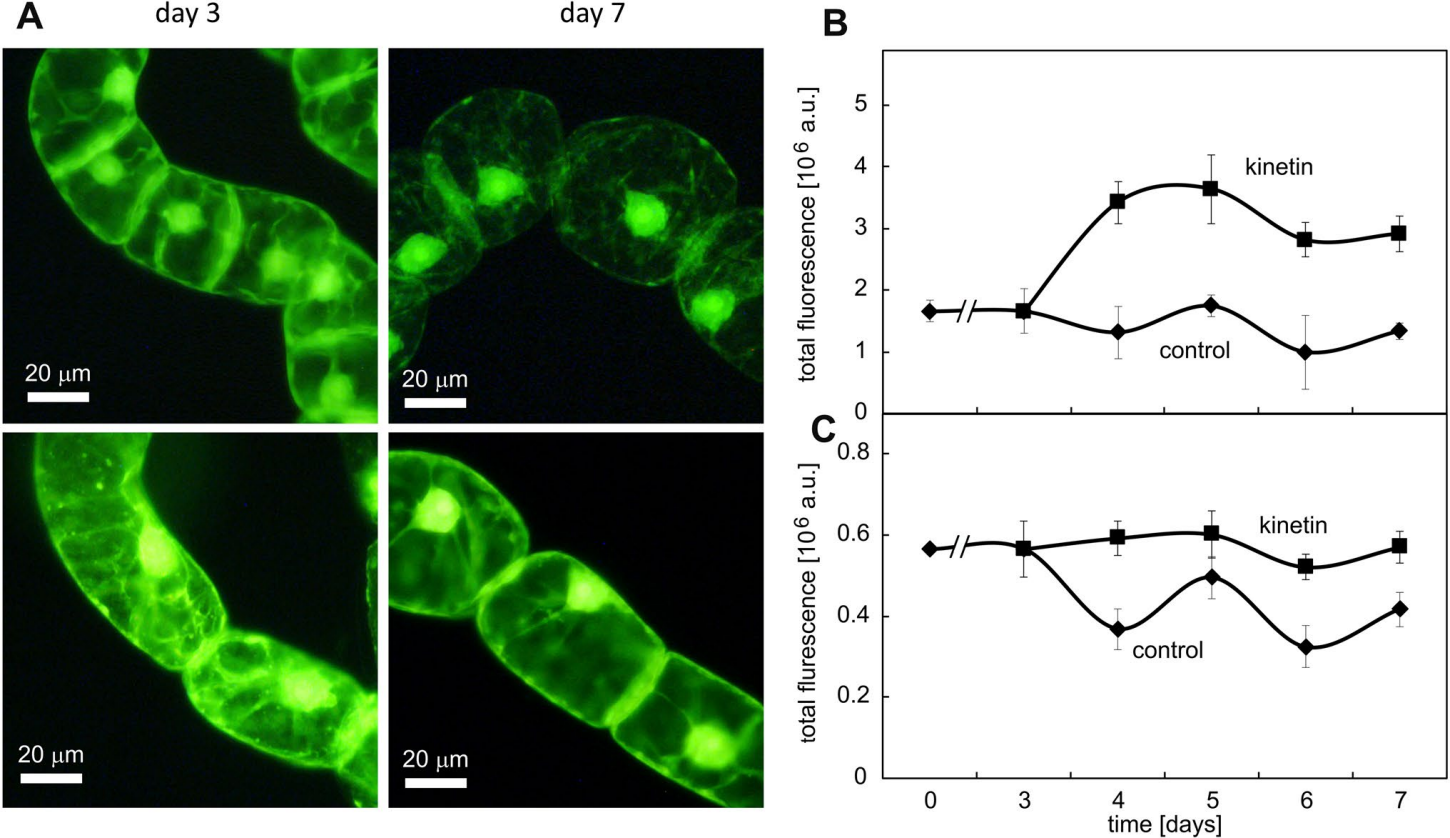
Effect of kinetin on intracellular calcium levels reported by chloro-tetracycline. Representative cells are shown in **A**, either at the peak of mitotic activity (day 3 after subcultivation) or at the end of the cultivation cycle (day 7 after subcultivation) either in control cells or cells treated with 50 µM kinetin from day 0. Quantification of the fluorescent signal in whole cells (**B**) and in nuclei (**C**). Data represent mean and standard errors from two independent experiments consisting of three technical replications with 50 to 100 individual cells and nuclei per replication

Regulated cell death in plants is often heralded by an increase of cytosolic calcium levels (reviewed in Huysmans et al. 2017). For instance, an increase of calcium is driving the activation of metacaspases executing autolysis in *Arabidopsis* (Watanabe and Lam 2011). We visualised, therefore, intracellular calcium using chloro-tetracycline through the entire cultivation cycle (Suppl. Fig. S3). In proliferating control cells, at day 3 after subcultivation, calcium was found in cytoplasmic strands, the nucleus and close to the cell walls, especially in the cross walls, where neighbouring cells are interconnected by plasmodesmata (Fig. 6A, top row). Later, at day 7 after subcultivation, the signal condensed to the nucleus, such that cytoplasm and cell walls were depleted from the signal. In response to kinetin, the fluorescence was significantly increased, not only in proliferating cells, but also in the stationary cells (Fig. 6A, bottom row). This impression was confirmed by the quantification of cytosolic and nuclear calcium ions (Fig. 6B and C). The cytoplasmic calcium signal in kinetin-treated cells increased drastically to around twice the level seen in the control, and whilst the signal gradually dissipated over time in both cases, this twofold elevation persisted until the end of the cultivation cycle (Fig. 6B). For the nuclear signal, the difference was seen as well, but it was less pronounced. In the control, fluctuations of calcium were observed with troughs at days 4 and 6 that were absent from the kinetin-treated cells (Fig. 6C). The increased level of intracellular calcium might derive from a stimulation of calcium uptake from the medium. The fact that around 20% more calcium remains in the supernatant in response to kinetin as compared to the control (Fig. 1B) would speak against this possibility. However, considering that FM is decreased by 30% in the kinetin-treated cells (Fig. 1A), one can estimate that the uptake per cell is slightly (around 10%) increased in the presence with kinetin.

### Kinetin is suppressing the accumulation of callose at the cross walls

**Fig. 7:**
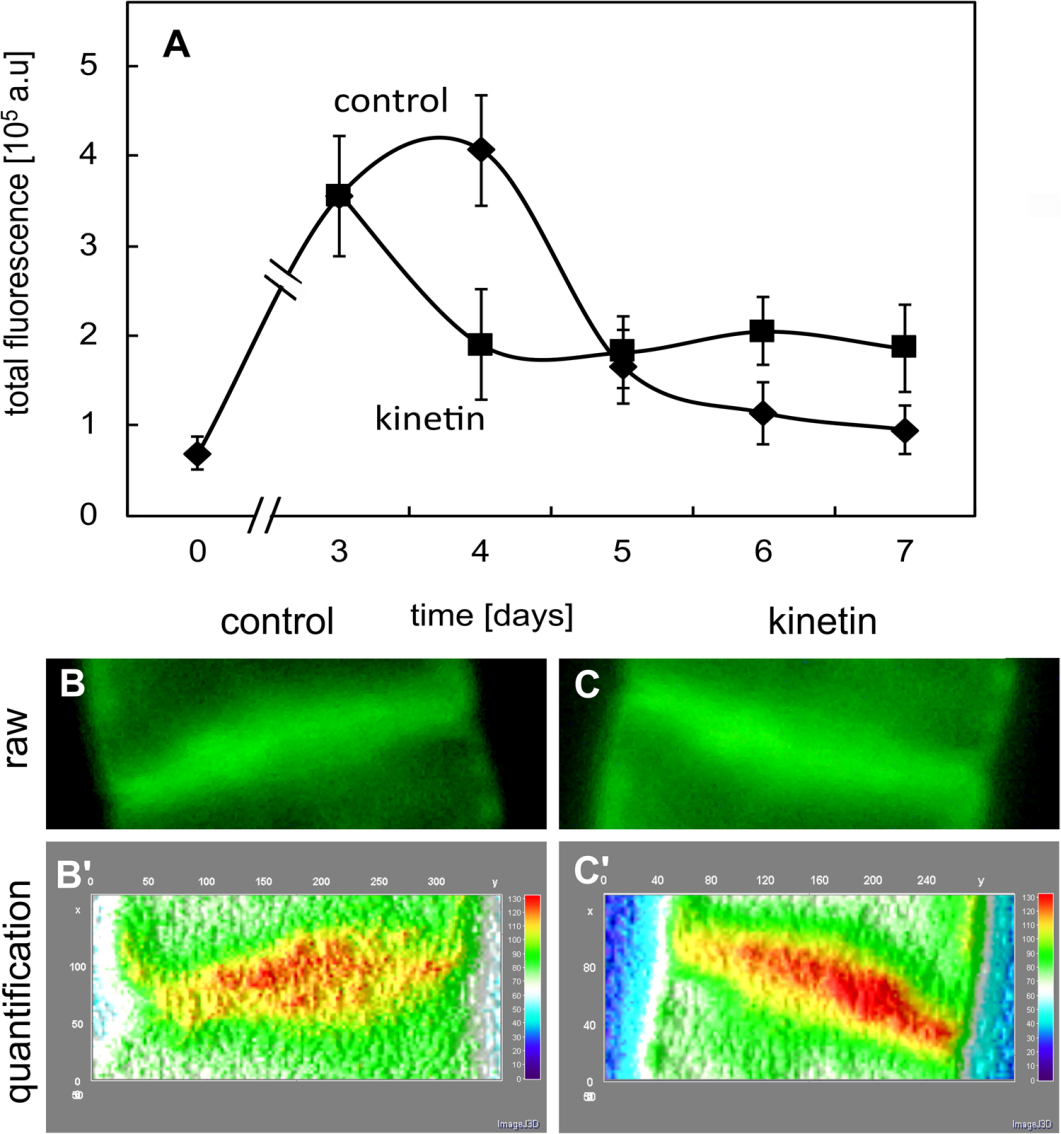
Effect of kinetin on callose abundance at the cross wall visualised by Aniline Blue. **A** Quantification of fluorescence in control cells from day 0 or in cells treated with 50 µM kinetin from day 3. Data represent mean and standard errors from two independent experiments consisting of three technical replications with a 150–250 individual cells per replication. **B** Representative images of the callose signal in control cells and **C** in kinetin-treated cells at day 7 after subcultivation. Heatmaps of signal abundance of callose, as yellow and red colour indicated by arrows, are shown in **B′** and **C′**, respectively. cw, cell wall

Plasmodesmata are crucial for cell–cell communication in plants, and in our previous work on kinetin-induced cell death of root cells (Doniak et al. 2017), we had observed that those cells that survived had deposited higher levels of callose at the plasmodesmata. This led us to visualise callose by Aniline Blue in tobacco BY-2 cells and to follow the signal over time in control cells and in cells treated with 50 µM of kinetin (Fig. 7). In control cells, the callose signal increased strongly, by a factor of > 4 until the end of the proliferation phase (day 4 after subcultivation and then dropped back swiftly to almost the initial level within 1 day) (Fig. 7A). In the kinetin-treated cells, this increase was less persistent and strongly dampened, reaching a plateau of around twofold from day 4 but was then sustained. As a result, the callose levels were higher in the kinetin-treated cells from day 6, because at that time the control cells had already returned to the initial (lower) level. The sharp peak of callose accumulation in proliferating control cells and the clear suppression and delay of this peak in cells treated with kinetin reflects the arrested cell cycles seen for the time course of DNA content (Fig. 3). At day 7 after subcultivation, the cross walls appeared brighter in the kinetin-treated cells (Fig. 7B). A quantification of fluorescence intensity in the cross wall confirmed an increase from around 150 ± 30 in the control cells to about 330 ± 50 in the kinetin-treated cells.

### Correlation analysis supports a central role of calcium and callose deposition

**Fig. 8:**
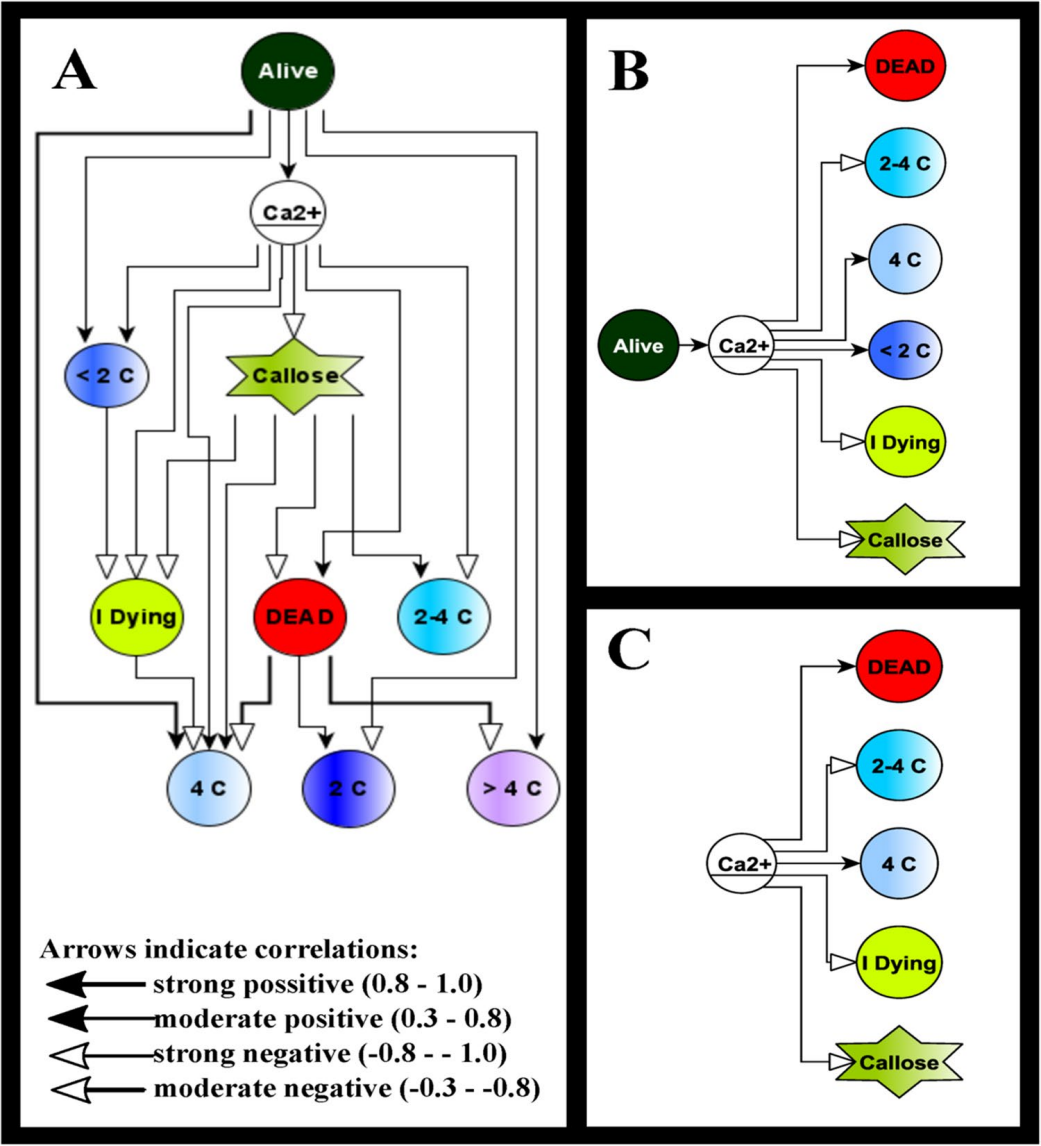
Graphical representation of Pearson correlation coefficients between cytosolic calcium (Ca^2+^), accumulation of callose, the different stages of cell death and the nuclear DNA content in response to kinetin in tobacco BY-2 cells

To facilitate identification of correlative patterns, we conducted a systematic analysis of Pearson correlation coefficients between all quantitative readouts, using a threshold of *P* < 0.05, correlating to a Pearson correlation coefficient above 0.3. The global correlation network (Fig. 8A) is placing cytosolic calcium ions and callose into the centre of the network. Within this network, two functional contexts emerge. On the one hand, the frequency of living, dead, hypoploid (C < 2*n*) and diploid cells correlates positively with calcium levels (Fig. 8B), whilst the amount of callose, the frequency of cells in stage I of dying at the first step and the frequency of *S* phase correlate negatively (Fig. 8B). The second functional network derives from the positive correlation of cells at C = 4*n* and of dead cells with calcium levels, whilst callose, the frequency of cells in stage I of dying and cells in *S* phase correlate negatively (Fig. 8C). Thus, calcium and callose change correlate inversely.

### Kinetin specifically disrupts microtubules and perturbs cell axiality

**Fig. 9:**
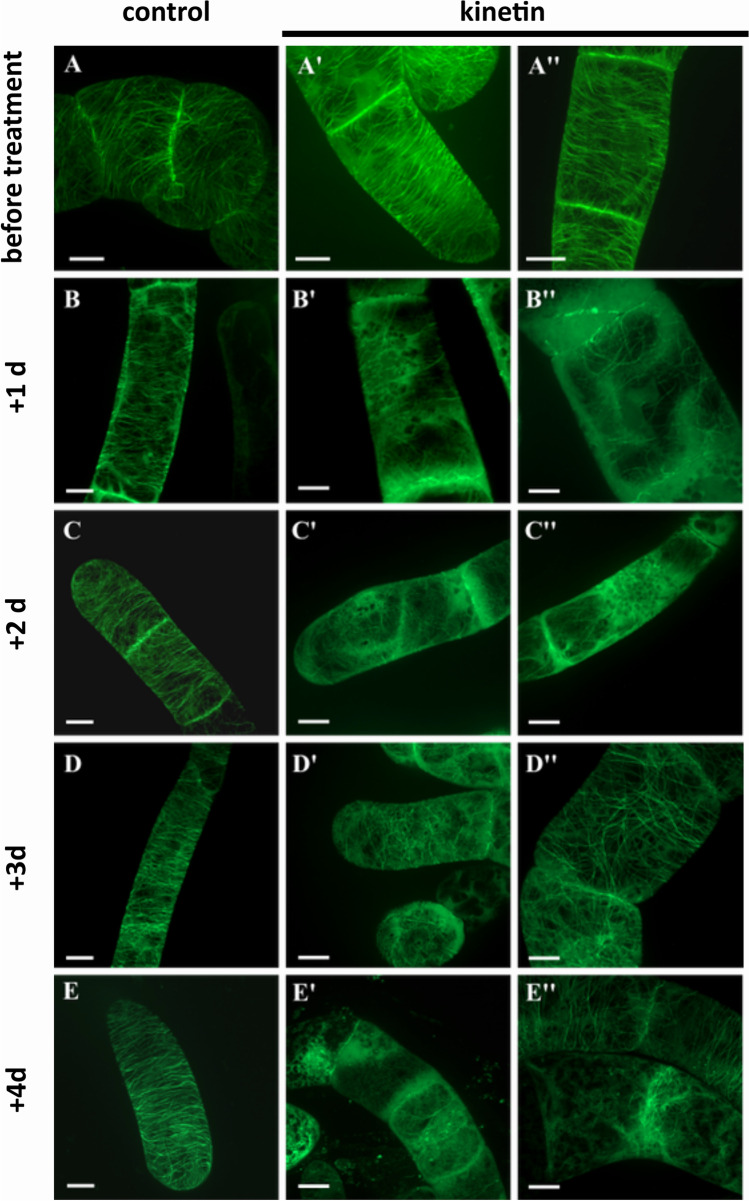
Effect of kinetin on microtubules visualised by the marker TuB6-GFP and followed by spinning-disc confocal microscopy through days 3 until 7. Fifty-micromolar kinetin was added at day 3. Scale bars 10 µm. The situation at day 3 prior to addition of kinetin is shown in **A-A″**. **B–E** Control cells and **B′–E′ and B″–E″** kinetin-treated cells

The cytoskeleton has been discussed to respond to signals that induce programmed cell death, whereby actin filaments are often linked with defence-related hypersensitive cell death (Chang et al., 2015), whilst microtubular reorganisation or disruption is characteristic for developmentally induced forms of cell death, such as the formation of xylem vessels (Iakimova and Woltering 2017). We followed, therefore, the response of microtubules in the tobacco BY-2 marker line TuB6-GFP (Hohenberger et al. 2011) to a treatment with kinetin at day 3 after subcultivation (Fig. 9). At this time, most cells had already completed their proliferation phase, such that axial cell files consisting of up to 6–8 cells were prevalent. Cortical microtubules were organised in bundles adjacent to the plasma membrane. The extent of their alignment was variable, depending on the progression into the expansion phase. In cells that were just in the final phase of cross-wall development (Fig. 9A), microtubules of different orientation co-existed in one cell. In contrast, cells already initiating expansion were characterised by parallel microtubules oriented perpendicular to the axis of expansion (Fig. 9A′, A″). The transverse orientation was most developed in terminal cells of a file (Fig. 9A′). In the absence of kinesin, these transverse arrays of parallel microtubules persisted until the end of the culture cycle (Fig. 9B–E). In response to kinetin, the microtubular cytoskeleton showed strong and time-dependent changes. One day following the addition of kinetin, most microtubules had disappeared (Fig. 9B′, B″). Only few remnants could be detected that were not aligned with the cell axis and often curved. This was accompanied by a strong increase of diffuse fluorescence in the cytoplasm, indicative of a high level of non-assembled tubulin heterodimers. At day 2 after addition of kinesin, this diffuse fluorescence was pronounced even more (Fig. 9C′, C″); it was almost impossible to find even remnants of microtubules. The diffuse GFP signal was most abundant at the cross walls, but also in the cell centre around the nucleus. Apparently, microtubules recovered during later time points, because at day 3 after addition of kinetin, partial arrays became detectable again (Fig. 9D′, D″), albeit lacking the preferential orientation prevalent in untreated control cells. Even 1 day later, equatorial arrays of microtubules were observed around the nucleus resembling a preprophase band (Fig. 9E′, E″).

**Fig. 10:**
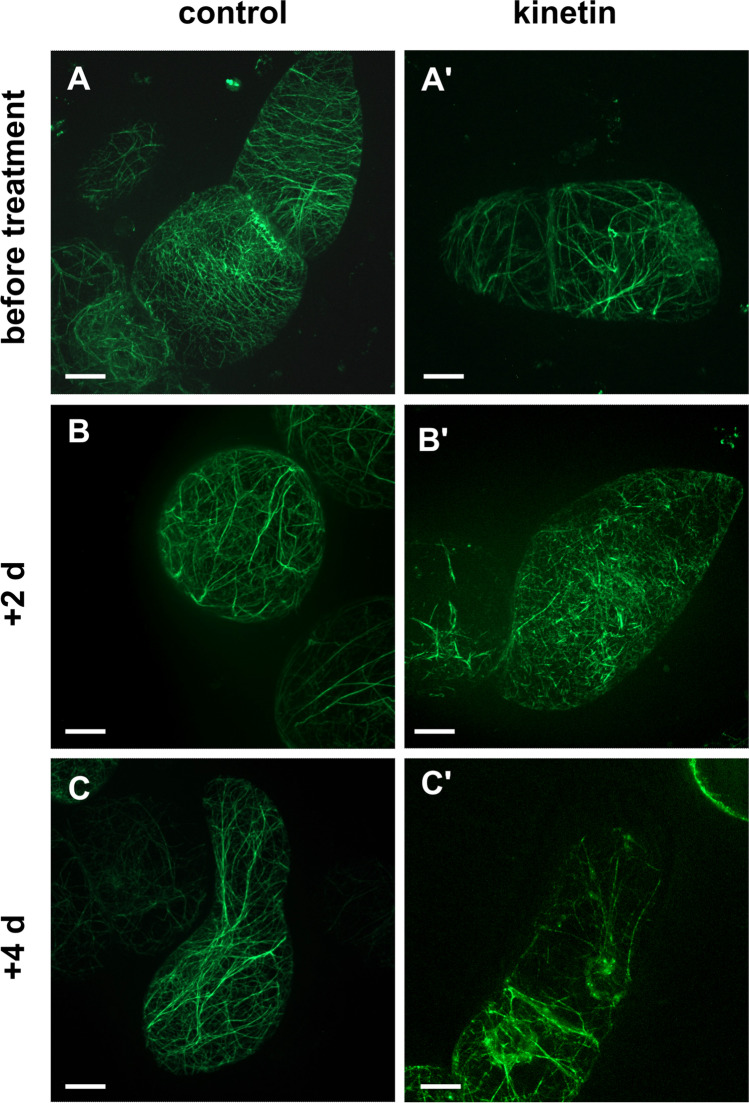
Effect of kinetin on actin filaments visualised by the marker FABD2-GFP and followed by spinning-disc confocal microscopy through days 3 until 7. Fifty-micromolar kinetin was added at day 3. Scale bar in *A* = 10 µm is applied to all images. **A** and **A″** The situation at day 3 prior to addition of kinetin in. **B–E** Control cells and **B′–E′** and **B″–E″** kinetin-treated cells

As to find out whether the effect of kinetin was specific for microtubules or whether it originated from a general breakdown of the cytoskeleton, we also probed actin filaments, using the tobacco BY-2 marker line GF11 (Voigt et al. 2005) expressing the actin-binding domain 2 from plant fimbrin in fusion with GFP (FABD2-GFP). In control cells, actin underwent a remodelling from a fine cortical meshwork in the cortical cytoplasm prevalent in proliferating cells (Fig. 10A, A′) towards the formation of transvacuolar actin cables, when the cells shifted to the expansion phase (Fig. 10B), which was also accompanied by a depletion of cortical actin (Fig. 10C). Under the influence of kinetin, this transition seemed delayed, because some cells with a rich cortical meshwork could be observed even at day 2 and day 3 (Fig. 10B′) following addition of kinetin. Only at day 4 (Fig. 10C′), the transvacuolar actin cables became prevalent. Thus, although actin filaments were responsive to kinetin, they remained intact; we did not note any indication for a disruption or elimination. Thus, the cytoskeletal effect of kinetin is specific for microtubules.

## Discussion

The motivation behind the current study was to get insight into the cellular mechanism of kinetin-induced cell death, a form of development-related PCD that was first described for the cortex cells of apical part of *V. faba* spp. *minor* seedling roots (Kunikowska et al. 2013; Doniak et al. 2014).

These findings lead to the following questions: (i) Does the kinetin response meet the criteria for a developmental PCD? (ii) What can we deduce from the correlative network connecting the different features of this phenomenon? (iii) What might be the role of the cytoskeleton in the elicitation or execution of PCD?

### The response to kinetin meets hallmarks of a developmental PCD

The response of BY-2 cells to kinetin meets criteria of a developmental PCD. First, several days are required until the death response becomes manifest, which is not compatible with acute toxicity. Second, by double staining with Orange Acridine and Ethidium Bromide, we can follow the loss of plasma-membrane integrity and the permeabilisation of the nuclear envelope (Fig. 4) and in a certain fraction of cells, the in stage II of dying, nucleolus breaks down (is negligible, indicating that the karyoplasm still maintains its structure until the end). The progressive disintegration of cellular membranes, not as unspecific consequence of cellular breakdown, but as upstream event, belongs to the main hallmarks of RCD (van Doorn 2011; van Doorn et al. 2011; Doniak et al. 2014; Kaźmierczak et al. 2017; Galluzzi et al., 2018). A further hallmark linked with RCD are aberrations of cell cycling. Nuclear DNA content (Fig. 3) suggests that kinetin arrests the cell cycle in the *S* phase. In response to the arrest of the cell cycle and the loss of membrane integrity, we observed a drastic increase in cytosolic calcium (Fig. 6), which seems to transduce the kinetin signal upon cellular responses.

The induction of cytosolic calcium during cytokinin-dependent cell death is consistent with findings in tobacco BY-2 cells, where inhibition of calcium influx by lanthanum ions mitigated cell death in response to hydrogen peroxide (Bobal et al. 2015). Conversely, the development of leaf perforations in the lace plant, a unique system to study developmental PCD, was promoted by the calcium ionophore A23187 and inhibited by the calcium channel inhibitor Ruthenium Red (Fraser et al. 2020). There is one important difference, though. In those systems, the increase of cytosolic calcium seems to derive from influx through the plasma membrane, which does not seem to be the case for kinetin induced cell death. Since the uptake of calcium per cell from the medium is inhibited (Fig. 1A, B) by kinetin, this increase of cytosolic calcium must derive from internal stores and not from stimulated influx. A study on kinetin-induced cell death in the *V. faba* ssp. *minor* system (Kaźmierczak et al. 2021) showed an increase of cytosolic calcium, which was accentuated by Ruthenium Red, suggesting that this calcium originates from internal stores, which is consistent with the current study. However, in the *V. faba* system, the elevated cytosolic calcium in presence of Ruthenium Red was accompanied by reduced cell death, which was also seen in the lace plant (Fraser et al. 2020). Thus, a bulk increase of cytosolic calcium does not per se lead to more pronounced cell death. Thus, specific subpools of calcium seem to be relevant that differ with respect to their sensitivity to Ruthenium Red. Possible mechanisms might be the activation of calcium-dependent nucleases, as found for the lysogenic formation of oil cavities in *Citrus* (Bai et al. 2020), or the activation of class I metacaspases that execute cell death (van Midden et al. 2021). The finding that isopentenyl adenosine induced cell death in BY-2 cells is mitigated by inhibitors of caspase activity (Mlejnek and Procházka 2002) rather points into the second direction, although the two mechanisms might act in concert.

In our tobacco cells, the increase in calcium is accompanied by a suppression of callose deposition that, in control cells, accompanies cell proliferation (Fig. 7). The antagonistic relationship between calcium and callose observed in our study (Fig. 8C) is consistent with observations in a mutant in *A. thaliana* which is overly sensitive to low calcium and functionally null for callose synthesis (Shikanai et al. 2020). An inverse correlation between callose and cell death has also to be concluded from findings in root cortex cells in *V. faba* ssp*. minor* that did not respond to kinetin by PCD, whilst callose deposition at their plasmodesmata was increased (Doniak et al. 2017).

Thus, the kinetin response of tobacco BY-2 cells to exogenous kinetin displays very specific features of developmental PCD and it even preserves details of the functional context between these features, such as the antagonistic behaviour of cytosolic calcium and callose deposition. It is therefore straightforward and justified to describe this phenomenon as RCD.

### What is the functional context of calcium, callose and cell cycle?

Calcium influx has been demonstrated for numerous cytokinin responses, starting from the classical work in mosses, where this phenomenon is integrated into the formative asymmetric division of caulonema cells required to define a bud (Saunders and Hepler 1983). Cytokinins can deploy a histidine-kinase-dependent two-component system (for a classical review see Hutchison and Kieber 2002). Using suspension cell cultures derived from respective mutants, the receptor HK4 was found to be central for cytokinin-induced PCD (Vescovi et al. 2012). Based on the ligand affinity patterns of different cytokinins, this receptor was also suggested to be involved in kinetin-induced cell death in *V. faba* ssp. *minor* roots (Kaźmierczak et al. 2021). Generally, cytokinin receptors deploy signalling through mobile response regulators, and some of those connect with G-protein signalling (Wang et al. 2017), which also would explain findings, where overexpression of a trimeric G-protein leads to a higher sensitivity against cytokinins (Plakidou-Dymock et al. 1998). G-protein coupled receptors are well known to induce calcium influx (for review see Tuteja and Sopory 2008), especially in stress-related contexts, such as the membrane transducer COLD1 driving cold sensing in rice (Ma et al. 2015). Our observation that the level of cytosolic calcium is elevated in kinetin-treated cells (Fig. 6) concurs with the situation in root cortex cells of *V. faba* ssp*. minor* (Doniak et al. 2016, 2017) and leads to the interesting question, whether this link is due to cytokinin-triggered activation of G-protein signalling.

The induction of cytosolic calcium can activate ethylene synthesis. A possible mechanism might be the activation of calcium-dependent protein kinases as CPK16 (Huang et al. 2013), phosphorylating ACC synthase at specific serine residues, such that it will remain protected from proteolytic decay. As a result, this enzyme will synthetise more of the direct ethylene precursor 1-aminocyclopropane-1-carboxylic acid (ACC). In fact, cytokinin treatment has been shown to maintain the activity of this enzyme (Chae et al. 2003). Ethylene is a central activator of PCD (for review see Wojciechowska et al. 2018) and is crucial for cytokinin-induced developmental cell death (Doniak et al. 2017).

Callose is often discussed in the context of pathogen defence and seems to be a tool to establish and maintain borders (Jacobs et al. 2003). Analysis of the *massue* mutant in *A. thaliana* indicate that callose is required for cytokinesis, although it is replaced by cellulose, xyloglucans and pectins in the maturating cell wall (Thiele et al. 2009), whilst persisting around the plasmodesmata (Wu et al. 2018). The synthesis of callose to delineate borders might have been an acquisition of the ancestral line within the pteridophytes that gave rise to the seed plants (Drábková and Honys 2017), but precursors might be even older, because the functional link between callose and cytokinesis is also present in the *Zygnematophyceae*, a sister clade of the terrestrial plant lineage (Davis et al. 2020). Our finding that callose intensity increases more than twofold in response to kinetin is congruent with a role of callose as a barrier between living and dying cells, a phenomenon that had already been reported for male sex differentiation in gametophytes of *Anemia phyllitidis* (Kaźmierczak 2008).

The missing link between elevated calcium, suppressed callose deposition and block of the cell cycle might be microtubules, since they can be eliminated through modulation of calcium- and calmodulin-binding associated proteins (Kölling et al. 2019). Elimination of microtubules in response to calcium would culminate in disrupted cytokinesis and should lead to binuclear or even multinuclear cells. This indicates that the suppression of callose is not upstream of microtubule elimination, but rather should be seen as a parallel phenomenon triggered by a common cause, e.g. the increase of cytosolic calcium. The fluctuations of nuclear calcium in control cells with troughs at day 4 and day 6 (Fig. 6C) are a mirror image of the incidence of cells in *G*_1_ (Fig. 3A), indicative of a role for nuclear calcium in *G*_2_. An implication of this discussion would be that kinetin, which is reducing the proportion of cells in *G*_1_ (Fig. 3B), should yield an elevated level of nuclear calcium, what is, in fact, observed (Fig. 6C).

Kinetin was originally discovered by its effect of mitosis in onion root tips (Guttman 1956). It promotes the progression through the *G*_2_-*M* checkpoint through activation of a tyrosine kinase that phosphorylates the p34^cdc2^ histone 1 kinase, which as a result becomes inhibited (tobacco cells; Zhang et al. 1996). In animal cells, mitotic arrest is often followed by apoptotic cell death (for reviews see Castedo et al. 2004; Kastan and Bartek 2004), and important anti-cancer compounds such as paclitaxel (Jordan et al. 1996) act through inducing this so-called mitotic catastrophe, often linked with multipolar spindles (Roninson et al. 2001) and their centriole clustering. Gallic acid that can block this clustering can specifically interfere with the proliferation of cancer cells (Tan et al. 2015). To what extent mitotic catastrophe exists in plants, is unclear. In a previous study, we have observed in tobacco BY-2 cells that cadmium can cause a death response displaying apoptotic features such as DNA laddering, if administered at the *G*_2_-*M* transition, whilst cells in *G*_1_ are dying in a necrotic fashion (Kuthanová et al. 2008). This would indicate that death in response to mitotic arrest also occurs in plants. Our finding that the cell death in response to kinetin is accompanied by an increased frequency of nuclei with lower DNA content would be consistent with a scenario, where the cell cycle is suppressed, which would then be followed by cell death.

In animals, apoptotic death in response to mitotic arrest is a caspase-independent mechanism using the activation of endonuclease G, followed by DNA fragmentation (Castedo et al. 2004). A link between kinetin and DNA damage has been proposed for the cortex cells in *V. faba* ssp*. minor* seedling roots (Doniak et al. 2014; Kaźmierczak and Soboska 2018). Moreover, kinetin can be formed as product from DNA oxidation in vivo (Barciszewski et al. 1997) and, thus, might act as signal reporting for DNA damage, initiating cell death to prevent damaged cells from proliferating.

### Microtubules versus actin: does the cytoskeleton decide the type of death?

The thorough elimination of microtubules followed by a complete loss of cell axiality (Fig. 9) belongs to the most striking cellular kinetin responses observed during the current study. This microtubule elimination is not an unspecific consequence of ensuing cell death, because at the same time, actin filaments as second important component of the cytoskeleton remain intact (Fig. 10). This delineates kinetin-induced cell death from defence-related cell death, where subcortical actin subtending the plasma membrane is rapidly degraded (Chang et al. 2015). Does this mean that the two ways to die use different components of the cytoskeleton as weapon to execute cell death?

Our observation stimulates three questions: (i) How can kinetin cause microtubule elimination? (ii) How is microtubule elimination causally linked with cell death? (iii) Which upstream event decides upon the cytoskeletal target to be dismantled?

The elimination of microtubules might well a consequence of the elevated calcium levels induced by kinetin (Fig. 6). A straightforward hypothesis would imply that calmodulin-related proteins (for review see Kölling et al. 2019) modulate the activity of microtubule-associated proteins. In fact, several members of the IQD clade of calmodulin-binding proteins have been shown to convey calcium-triggered subdomains to microtubules (Bürstenbinder et al. 2017). The resulting breakdown of the microtubular cytoskeleton would lead to mitotic catastrophe and deploy cell death. Alternatively, calcium might activate metacaspases (Zhu et al. 2020) that could break down microtubule-associated proteins — in analogy to the breakdown of neural MAP *τ* by caspase 3 (Fasulo et al. 2000). The situation is different for actin filaments. Here, it is the membrane located NADPH oxidase Respiratory burst oxidase Homologue that is responsible for the breakdown of the cortical actin network (Chang et al. 2015). Both apoplastic oxidative burst and calcium influx are important signals conveying information about different stress conditions. They can occur with different timing and balance and this temporal signature has been shown to activate different qualities of defence responses in grapevine cells (Chang and Nick 2012). It is, therefore, worth to address the function of the cytoskeleton in kinetin-induced cell death using inhibitors blocking calcium influx, apoplastic oxidative burst and the two components of the cytoskeleton, actin filaments and microtubules. The possibilities outlined above can be used to infer testable implications and get deeper insight into the role of the cytoskeleton for steering cell death. It will also be rewarding to use tobacco cell lines, where specific classes of metacaspases have been overexpressed to dissect the molecular mechanism behind this phenomenon.

## Supplementary Information

Below is the link to the electronic supplementary material.

Suppl. Fig. S1 **A’-F’** Representative images showing the response of tobacco BY-2 cells to 50 µM kinetin as compared to **A-F** non-treated, control, at different days after sub-cultivation after staining with DAPI. Size bar is 50 µm. (PDF 232 KB)

Suppl. Fig. S2 **A-F** Fluorescence intensity histograms and **A’-F’** inferred frequency distributions of nuclear DNA content in **A, A’** control cells and **B-F, B’-F’** cells treated with 50 µM kinetin sampled at different days after sub-cultivation (PDF 97 KB)

Suppl. Fig. S3 Representative images showing the fluorescent calcium reporter chloro-tetracyclin reporting intracellular calcium levels in tobacco BY-2 cells **A’-F’** treated with 50 µM kinetin as compared to **A-F** non-treated controls () at different days after sub-cultivation. Size bar is 50 µm. (PDF 285 KB)

## Data Availability

The datasets presented in this study are stored on the server of the Steinbuch Centre for Computing and are made available on reasonable request.
