# Supplementary figures and images for: Kinetin induces microtubular breakdown, cell cycle arrest and programmed cell death in tobacco BY-2 cells
Source: Protoplasma. 2022 Oct 14;260(3):787–806. doi: 10.1007/s00709-022-01814-6 (PMC10125952; doi:10.1007/s00709-022-01814-6)

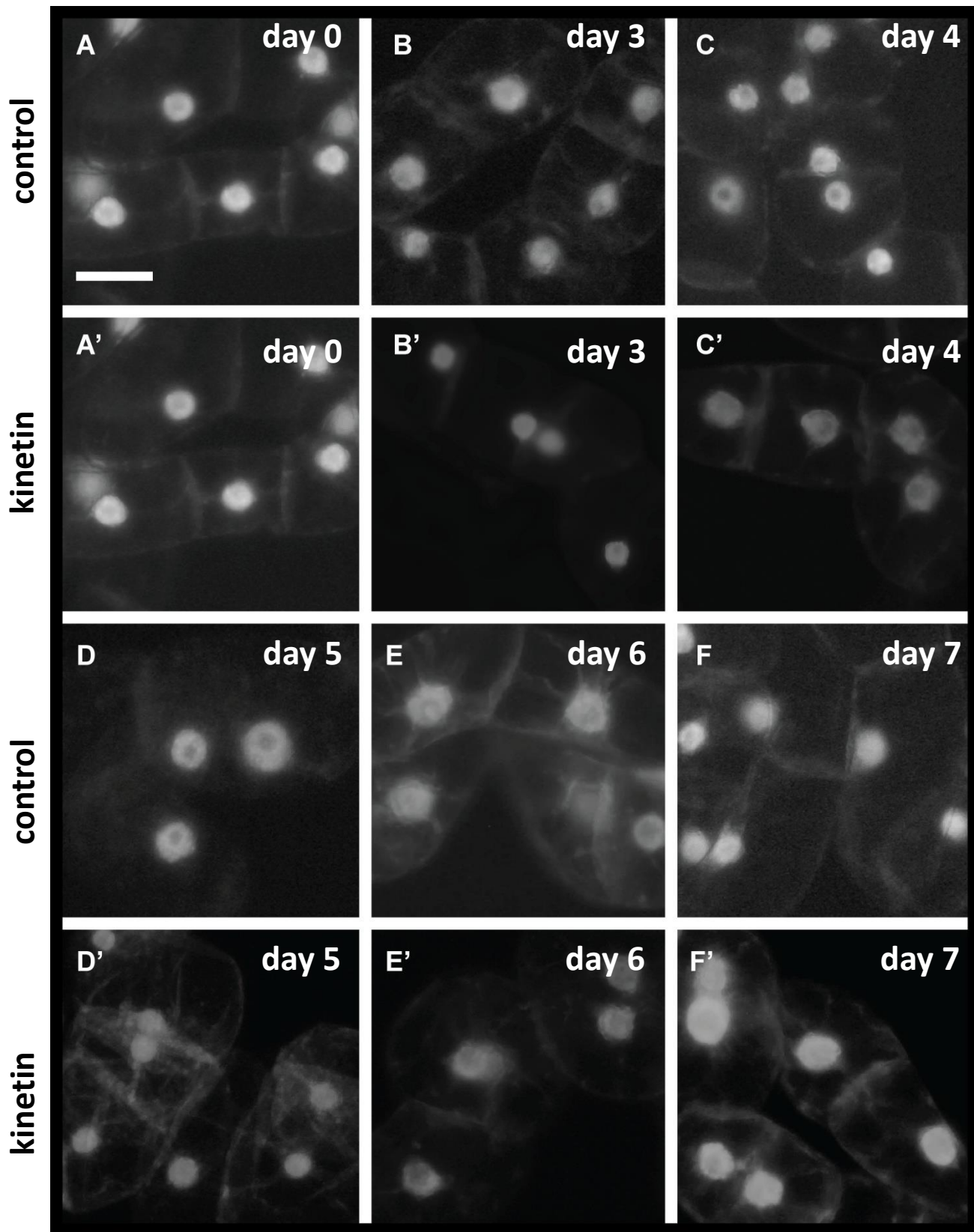

Supplement: Supplementary file 1 — A’-F’ Representative images showing the response of tobacco BY-2 cells to 50 µM kinetin as compared to A-F non-treated, control, at different days after sub-cultivation after staining with DAPI. Size bar is 50 µm. (PDF 232 KB) [file 709_2022_1814_MOESM1_ESM.pdf]

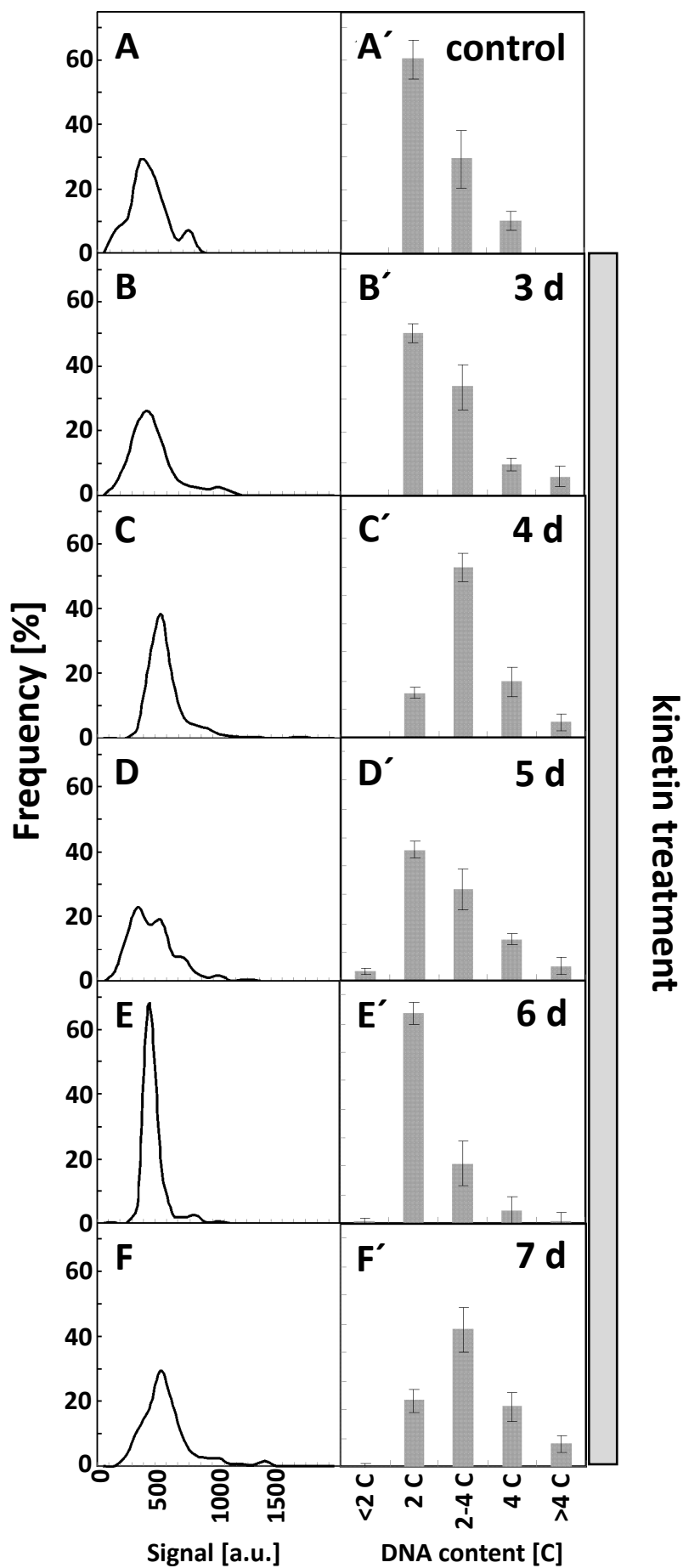

Supplement: Supplementary file 2 — A-F Fluorescence intensity histograms and A’-F’ inferred frequency distributions of nuclear DNA content in A, A’ control cells and B-F, B’-F’ cells treated with 50 µM kinetin sampled at different days after sub-cultivation (PDF 97 KB) [file 709_2022_1814_MOESM2_ESM.pdf]

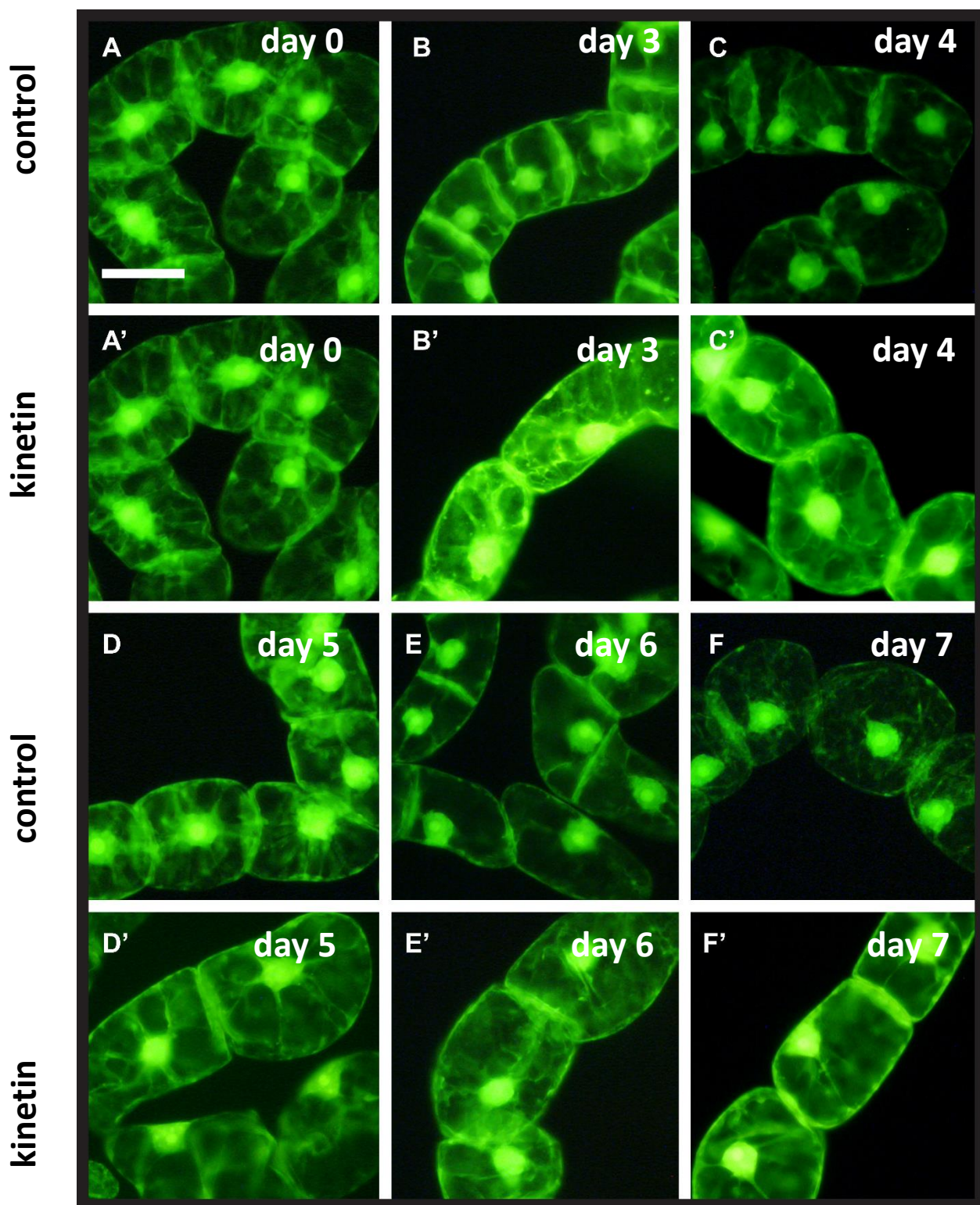

Supplement: Supplementary file 3 — Representative images showing the fluorescent calcium reporter chloro-tetracyclin reporting intracellular calcium levels in tobacco BY-2 cells A’-F’ treated with 50 µM kinetin as compared to A-F non-treated controls () at different days after sub-cultivation. Size bar is 50 µm. (PDF 285 KB) [file 709_2022_1814_MOESM3_ESM.pdf]
